# Systematic review of management strategies to control chronic wasting disease in wild deer populations in North America

**DOI:** 10.1186/s12917-016-0804-7

**Published:** 2016-08-22

**Authors:** F. D. Uehlinger, A. C. Johnston, T. K. Bollinger, C. L. Waldner

**Affiliations:** 1 Large Animal Clinical Sciences, Western College of Veterinary Medicine, University of Saskatchewan, 52 Campus Drive, Saskatoon, S7N 5B4 Canada; 2 Canadian Wildlife Health Cooperative, Department of Veterinary Pathology, Western College of Veterinary Medicine, University of Saskatchewan, 52 Campus Drive, Saskatoon, S7N 5B4 Canada

**Keywords:** Chronic wasting disease, Control, Wild deer, North America

## Abstract

**Background:**

Chronic wasting disease (CWD) is a contagious, fatal prion disease affecting cervids in a growing number of regions across North America. Projected deer population declines and concern about potential spread of CWD to other species warrant strategies to manage this disease. Control efforts to date have been largely unsuccessful, resulting in continuing spread and increasing prevalence. This systematic review summarizes peer-reviewed published reports describing field-applicable CWD control strategies in wild deer populations in North America using systematic review methods. Ten databases were searched for peer-reviewed literature. Following deduplication, relevance screening, full-text appraisal, subject matter expert review and qualitative data extraction, nine references were included describing four distinct management strategies.

**Results:**

Six of the nine studies used predictive modeling to evaluate control strategies. All six demonstrated one or more interventions to be effective but results were dependant on parameters and assumptions used in the model. Three found preferential removal of CWD infected deer to be effective in reducing CWD prevalence; one model evaluated a test and slaughter strategy, the other selective removal of infected deer by predators and the third evaluated increased harvest of the sex with highest prevalence (males). Three models evaluated non-selective harvest of deer. There were only three reports that examined primary data collected as part of observational studies. Two of these studies supported the effectiveness of intensive non-selective culling; the third study did not find a difference between areas that were subjected to culling and those that were not. Seven of the nine studies were conducted in the United States.

**Conclusions:**

This review highlights the paucity of evaluated, field-applicable control strategies for CWD in wild deer populations. Knowledge gaps in the complex epidemiology of CWD and the intricacies inherent to prion diseases currently pose significant challenges to effective control of this disease in wild deer in North America.

**Electronic supplementary material:**

The online version of this article (doi:10.1186/s12917-016-0804-7) contains supplementary material, which is available to authorized users.

## Background

Chronic wasting disease (CWD) is a contagious prion disease affecting free-ranging mule deer (*Odocoileus hemionus*), white-tailed deer (*Odocoileus virginianus*), elk (*Cervus elaphus*) and moose (*Alces alces*) in a growing number of regions across North America. To date, control efforts have been unsuccessful, resulting in continuing geographic spread and increasing prevalence in endemic zones. Projected deer population declines and concern about the potential spread of CWD to other species warrant strategies to manage this disease.

Chronic wasting disease has a long incubation period [1], during most of which deer transmit the disease and contribute to environmental contamination via shedding prions in saliva [2, 3], urine [4, 5], feces [6, 7], and velvet [8], as well as through infected carcasses [9–11]. Chronic wasting disease is spread through either direct animal contact or prion contaminated environments [2, 12]. Soil is an environmental reservoir for prion infectivity [13]. Chronic wasting disease prions in the environment can remain available and infectious for at least 2.5 years [10], and scrapie prions in soil have been shown to be infectious after 16 years [14]. The combination of direct and environmental transmission of CWD prions makes it very difficult to control this disease in wild populations. Neither licenced vaccines nor therapies are currently available. Therefore, population control, primarily through nonselective hunting and culling of deer, has been the most widely attempted management strategy with mixed results [15]. Some states have moved their goal of CWD eradication to one of CWD containment [16, 17]. Strategies to prevent further spread include carcass regulations, restrictions on importing and raising captive cervids, and bans on feeding and baiting practices.

The primary objective of this study was to identify, summarize and evaluate published reports describing field-applicable CWD control strategies in wild deer populations in North America using structured and transparent review methods. The secondary objectives were to identify key knowledge gaps and discuss the available evidence and its limitations.

## Methods

### Approach to literature synthesis and team

A systematic review methodology was applied to search and evaluate the literature [18]. All four co-authors were involved in the identification and appraisal of the literature as outlined below, contributing diverse methodological and subject expertise.

### Review question, eligibility criteria and search strategy – peer-reviewed literature

The methods followed to conduct this review were summarized by Sargeant and O’Connor [18]. This review is restricted to the information reported in the peer-reviewed literature. The PRISMA Statement’s 27-item checklist for completing a systematic review was applied to the identified observational and predictive modeling studies [19]. The list is available as an additional file to this manuscript (see Additional file 1). The review question used to develop the literature search was: ‘What control strategies for chronic wasting disease have been evaluated in wild deer in North America and what evidence is there that the evaluated strategies resulted in reduced disease risk measured by CWD prevalence, geographic spread or wild deer population size?’. For peer-reviewed literature, ten databases were selected and searched because of their relevance to veterinary medicine and animal management: Medline, Embase Classic, CABI/Global Health, BIOSIS, Zoological Record, Web of Science Core Collection, AGRICOLA, BioONE, Animal Behavior, and Scopus. No limitations were placed on the date and all databases were searched up until May 2015. Where database search allowed, limits were applied to select only references written in English and conducted in North America. Table 1 showed an example search strategy.

**Table 1:** Example search strategy from OVID Platform: Scopus 1947 to 2015 Week 19

1	“chronic wasting disease”
2	“CWD”
3	“TSE” OR “transmissible spongiform encephalopathy”
4	Prion*
5	#1 OR #2 OR #3 OR #4
6	deer OR cervid*
7	6 AND NOT (farm* OR captiv* OR tame* OR experiment* OR laboratory OR “in vitro”)
8	#5 AND #7
9	Limit 8 to English AND (US OR CANADA)

A comprehensive search strategy was applied by initially combining the subject keywords (deer OR cervid*) with the exclusion keywords (e.g. (farm* OR captiv* OR tame*) and (experiment* OR laboratory OR “in vitro”), which enabled the selection of literature on wild deer populations; these were then combined with the disease terms (e.g. Chronic Wasting Disease OR prion* OR transmissible spongiform encephalopathy) to identify further articles. All references identified through these searches were imported into EndNote X7 (Thomson Reuters, Philadelphia, USA) for screening and deduplication. Duplicates were first removed from each individual database group, and then removed from the whole library, ensuring that each database group contained a unique set of articles, and that no article was present in more than one database group. A table with the number of unique articles collected from each database is available as an additional file (see Additional file 2). Following deduplication, 799 unique articles were exported to Microsoft Office Access (2013) (see Additional file 2). Fifty-one were immediately eliminated because they were not written in English. The remaining 748 were retained in the database for relevance screening.

### Relevance screening

Three reviewers (ACJ, FDU, CLW) screened the titles and abstracts of all 748 unique citations identified in the search. Specific relevance criteria were posed as five questions, to which a “yes” or a “no” answer for each abstract was given: (1) Is the literature in English? (2) Was the research based in North America? (3) Does the research include wild deer? (4) Does the research include chronic wasting disease? (5) Does the research discuss control or management strategies? A single “no” resulted in exclusion of that article from the citation base. When a title or abstract did not include sufficient detail to assess the reference’s relevance, the citation was retained for further evaluation. Disagreements between reviewers were settled with discussion and consensus. From the list of articles that had a “yes” to question 5 (control), one author (ACJ) also reviewed the reference lists of the seven most recent articles and five additional studies were added to the pool of references to be screened for eligibility. Following this relevance screening, 61 references were identified.

### Data extraction

To extract relevant data and study characteristics, including potential for bias, from all identified references, a data collection form containing 38 main questions was developed. Data extracted included, but was not limited to the species of deer being studied, confirmation that the study was not focussed on a farmed or captive herd, a description of the control measure under study, and a description of the study design including a description of the control or reference population for the observational studies. Most questions required binary answers (yes/no); other questions required numerical (e.g. ‘year data collection begin’; ‘study population size’) or text (e.g. ‘study location’) entries specific to the study. Each main question gave the opportunity to select ‘other’ and insert an explanation in a text field. The biases and limitations of the individual observational studies reported by the authors were summarized in a table. The sources of uncertainty, assumptions and limitations of the predictive models reported by the authors and identified by the reviewers were also reported in summary tables and the text. In total, 185 data points were extracted from each study. Each full-text reference was examined independently using these questions by at least two authors (ACJ, CLW, FDU), ensuring consistency and accuracy. Discrepancies between two authors were resolved through discussion and consensus. Following detailed appraisal of each of these 61 full-text references, 14 articles remained where the objectives included evaluating control strategies for chronic wasting disease in wild deer in North America.

### Subject expert confirmation

A subject matter expert (TKB) was consulted and asked to cross-check the 14 selected articles and to identify any relevant but missing references. Two additional references were identified in this manner resulting in a total of 16 studies. In multiple panel sessions, three authors (TKB, CLW, FDU) re-visited each of these 16 articles to confirm their relevance and accuracy of data extraction [20–35]. In this manner, four articles were subsequently excluded. Two of these articles addressed hunting behaviour or modifications of hunting season but did not explicitly investigate the effects on chronic wasting disease in wild deer [32, 33]. One article investigated the feasibility of a control practice but not its efficacy [34]. The fourth was a critique of published models and their application for chronic wasting disease in deer and elk [35]. Twelve references were ultimately available for data analysis.

### Grey-literature and publically available technical reports

While the objective of this review was not to summarize the grey-literature, we did a search to identify the potential that important information relevant to the study objectives could have been missed by focussing solely on peer-reviewed publications. To identify publically available technical reports national, provincial, and state government document databases, ProQuest Dissertations and Theses database, Open Access Theses and Dissertations database, and Theses Canada (Library and Archives Canada), along with key organizational websites related to wildlife disease were searched. The first 50 hits of Google search, Yahoo! search and those mentioned above were investigated. All searches conducted for grey literature used the keywords described for the peer-reviewed literature search. Identified resources were screened specifically with a focus on identifying new information relevant to CWD control which was not contained within the selected peer-reviewed literature, including relevant news items and fact sheets. The resulting Microsoft Excel spreadsheet with 45 active URLs is available from the authors on request. The listings were checked and links were updated on March 12, 2016.

### Data analysis

Data extraction from the peer-reviewed articles and descriptive analyses from the 185 data extraction points were conducted using Microsoft Excel. Key results were reported in frequency tables and figures. The analysis included summarizing whether authors described specific sources of uncertainty in the results of dynamic simulation studies, reported potential biases in descriptive or analytical studies, and whether authors described other limitations in the design or results of their study. As the review dealt with a small number of predictive modeling papers and observational studies and there was no consistent measure of effect reported for the observational studies, no attempt was made to conduct a meta-analysis of the effectiveness of the control measures. Rather, the control measures under investigation were described with relevant study findings summarizing the success of the control measure.

## Results

### Database search results and summary of reviewed articles

**Fig. 1:**
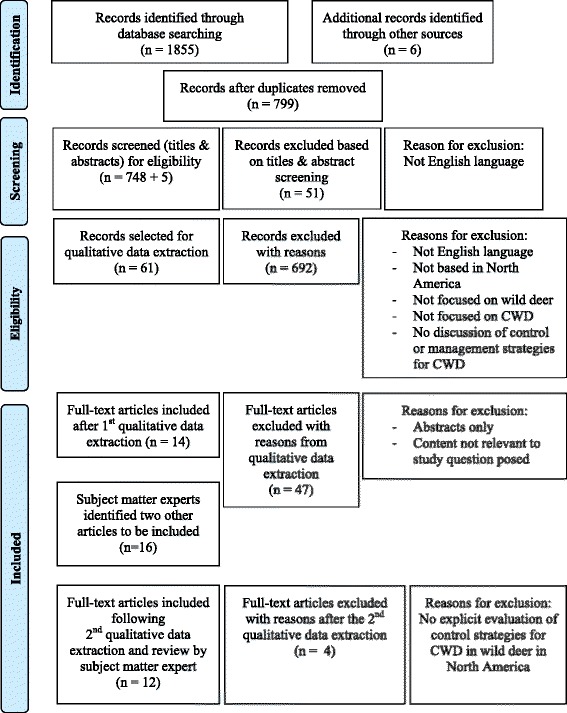
PRISMA diagram summarizing literature database search

A flow chart of the database search results and number of unique articles selected was depicted in Fig. 1. Table 2 summarized the characteristics and designs of the 12 primary studies selected for final review. The studies could be classified into three main study design categories: six of the 12 studies used predictive modeling to evaluate control strategies; three were analytical observational studies and threw were analytical experimental studies. Most (5/6) of the predictive modeling studies were deterministic [21–25], while only one was stochastic [20]. The predictive modeling studies used a combination of hypothetical scenarios [22, 23, 25], field observations [20–22, 24], experimental data [20, 22], and expert opinion [22] to parameterize the models.

**Table 2:** Description of 12 studies evaluating control strategies of CWD in wild deer in North America

Author and year of publication	Study location	Data collection period	Target population	Sex or age-specific analyses	Study design	Intervention	Outcome evaluated	Conclusions
Predictive modeling studies								
Gross and Miller, 2001, J Wildl Manage [20]	Colorado	NA	Mule deer	Sex (m/f)- and age-specific	Stochastic and agent-based predictive model	Selective culling and varying transmission rates (method undefined)	Change in CWD prevalence Change in wild deer population size Cost not evaluated	Control measures effective in some scenarios
Wasserberg et al., 2009, J Appl Ecol [21]	Wisconsin	NA	White-tailed deer	Sex (m/f)- and age-specific	Multi-state non-spatial deterministic predictive matrix model	Recreational harvest and additional population reduction for disease management	Change in CWD prevalence Change in wild deer population size Cost not evaluated	Control measures effective in some scenarios
Wild et al., 2011, J Wildl Dis [22]	National Park Service, Colorado^a^	NA	Not specified, wild deer	No	Deterministic predictive modeling using differential equations	Role of large predators [wolves] in disease control	Change in CWD prevalence Change in wild deer population size Cost not evaluated	Control measures effective in some scenarios
Potapov et al., 2012, Proc R Soc B [23]	University of Alberta, Alberta^a^	NA	White-tailed deer	Age-specific (adults only)	Deterministic, stock-and-flow predictive modeling	Increased overall hunting pressure with and without combination of vaccination	Change in CWD prevalence Change in wild deer population size Cost not evaluated	Control measures effective in some scenarios
Jennelle et al., 2014, PLOS One [24]	Wisconsin	NA	White-tailed deer	Sex (m/f)- and age-specific	Deterministic, compartmental predictive modeling	Increased overall hunting pressure; increased hunting pressure on targeted sex group	Change in CWD prevalence Extent of geographic spread of CWD Change in wild deer population size Cost not evaluated	Control measures effective in some scenarios
Oraby et al., 2014, J Theor Biol [25]	University of Ottawa, Ontario^a^	NA	Not specified, wild deer	No	Deterministic, stock-and-flow predictive modeling	Seasonal hunting	Change in CWD prevalence Change in wild deer population size Cost not evaluated	Control measures effective in some scenarios
Analytical observational studies								
Conner et al., 2007, Ecol Appl [26]	Colorado	1996 – 2005	Mule deer	Sex (m)- and age-specific	Analytical; observational, before-and-after control impact (BACI)	Planned culling (focal culling in hot spots using sharpshooters)	Change in CWD prevalence Cost not evaluated	Control measure not effective
Mateus-Pinilla et al., 2013, Prev Vet Med [27]	Illinois	2003 – 2008	White-tailed deer	Sex (m/f)- and age-specific	Analytical; observational; cohort	Planned culling (government-organized, localized culling, population control permits and nuisance deer removal permits using sharpshooters)	Change in CWD prevalence Cost not evaluated	Control measure effective
Manjerovic et al., 2014, Prev Vet Med [28]	Wisconsin; Illinois	2003 – 2012	White-tailed deer	No	Analytical; observational, cross-sectional	Planned culling (government-organized, localized culling using sharpshooters)	Change in CWD prevalence Cost not evaluated but acknowledged as a need	Control measure effective
Analytical experimental studies^b^								
Wolfe et al., 2012 [29]	Colorado; Research Facility	2003	Mule deer	~15 months at beginning of trial	Analytical; experimental trial; controlled	Oral administration of pentosane polysulfate; tannic acid; tetracycline HCl for prevention of CWD infection	Change in CWD status of individual deer	Control measure not effective
Pilon et al., 2013 [30]	Colorado; Research Facility	2007 – 2009	Mule deer	No	Analytical; experimental trial; controlled	Intramuscular vaccination with two different prion peptide sequences	Change in CWD status of individual deer	Control measure not effective
Goñi et al., 2015, Vaccine [31]	Colorado; Research Facility	Not reported	White-tailed deer	No	Analytical; experimental trial; controlled	Mucosal immunization with attenuated *Salmonella* vaccine expressing PrP	Change in CWD status of individual deer and length of incubation period	Efficacy of control measure not clear

^a^Location of modeling team. Location of population under study was not specified

^b^These studies were excluded from further review

Within the three observational studies, there was a cohort study [27], a cross-sectional study [28], and a meta-analysis of before-and-after control impact (BACI) data [26]. The cohort study examined the association between population management intervention pressure and change in CWD prevalence in Illinois from 2002 to 2008 based on 14,650 test results from white-tailed deer [27]. The cross-sectional study compared changes in CWD prevalence between Illinois and Wisconsin over a 10-year period that included 5 years when both states were actively culling and 5 years when active culling stopped in Wisconsin [28]. In the meta-BACI study, 16 areas of sustained treatment were compared to spatially paired control areas in Colorado from 2001 to 2005 [26].

The three controlled, experimental trials investigated potential future approaches to control of CWD in wild deer. As the purpose of this review was to assess field-applicable control strategies and their effects on CWD prevalence, geographic spread, or wild deer population size, the three experimental articles were excluded from subsequent analysis because there was insufficient information to evaluate the potential of these approaches for disease control in wild cervids at this time.

Seven of the remaining nine studies were conducted in the United States and published between 2001 and 2014 using data collected between 1996 and 2012 (Table 2). Two modeling studies were reported from Canada; one study examined CWD in white-tailed deer [23] and the other did not specify species [25] (Table 2). The target populations were mule deer in 2/9 (one analytical, one predictive modeling study) and white-tailed deer in 5/9 (two analytical, three predictive modeling studies) studies, while 2/9 (two predictive modeling studies) did not specify a target deer species (Table 2).

In the government documents, websites, and theses identified there were no obvious unique studies specifically related to the study objectives that had not been captured in the peer-reviewed literature (data not shown). Because this was not intended to be an exhaustive search, it is possible that documents or theses were missed that might contain new information; however, the objective of this report was to document the extent and limitations of the peer-reviewed literature.

### Control interventions and outcomes

The control interventions and the outcomes evaluated for each of the nine remaining studies were listed in Table 2. None of the nine papers examined the costs of the reported intervention. One of the papers specifically acknowledged the importance of this information [28]. Four interventions or control strategies were evaluated: selective or preferential removal of infected deer; general, non-selective population reduction (through hunting, intensive agency culling and/or harvest permits); change in season of harvest; and vaccination (Table 3).

**Table 3:** Effectiveness^*^ of 4 intervention strategies for CWD control in wild deer in North America

Study	Intervention strategy				
	Selective removal	Non-selective removal		Seasonal (summer) hunting	Vaccination
Predictive modeling studies					
Gross and Miller, 2001, J Wildl Manage [20]	Effective^a,b^				
Wasserberg et al., 2009, J Appl Ecol [21]		Effective			
Wild et al., 2011, J Wildl Dis [22]	Effective^c^				
Potapov et al., 2012, Proc R Soc B [23]		Effective^d^			Effective
Jennelle et al., 2014, PLOS One [24]	Effective^e^		Not effective		
Oraby et al., 2014, J Theor Biol [25]				Effective	
Analytical observational studies					
Conner et al., 2007, Ecol Appl [26]			Not effective		
Mateus-Pinilla et al., 2013, Prev Vet Med [27]		Effective			
Manjerovic et al., 2014, Prev Vet Med [28]		Effective			

^*^All models showed some degree of effectiveness depending on parameters and scenarios chosen. Effectiveness was defined based on the extent to which the specific management objectives were achieved or were projected to be achieved by the specific intervention. Most studies, depending on the specific objectives, were considered effective when the control measure either maintained CWD at low prevalence or reduced it to low or zero prevalence

^a^Preferential removal of infected deer

^b^Effective only when CWD prevalence was low (0.01 and 0.05) and when 80–90 % of infected deer could be removed after 80 years of intervention

^c^Preferential removal of infected deer by large predators

^d^Effective only when assuming a FD transmission of CWD but not when assuming a DD transmission

^e^Preferential removal of infected deer by targeting males which have a higher prevalence

Studies assessing selective removal of infected deer evaluated scenarios where this occurred with large carnivores [22], or by increased harvest of bucks which have a higher prevalence of CWD [24] or by test (varying from removing sick to detecting preclinical deer) and cull [20]. All studies evaluating selective removal did so using predictive models and all were effective under specific scenarios.

The second type of intervention could be summarized as non-selective herd reduction. This method was effective in predictive modeling studies depending on parameters chosen and the level of culling used in the scenarios [21, 23, 24]. Non-selective population reduction was the only management intervention that has been evaluated under field conditions in observational studies and in all three cases this involved government-organized sharpshooters removing additional animals beyond those removed during hunting seasons. One study showed no benefit on CWD prevalence [26] whereas the two other studies demonstrated an effect on reducing CWD prevalence compared to sites where hunting was the only method of population control [27, 28]. Seasonal (summer) hunting [25] and the use of vaccine [23] was shown to be effective again with predictive models under certain conditions.

### Individual study characteristics and outcomes

#### Non-selective culling

##### Predictive modeling studies

The evidence that increased overall hunting pressure has a beneficial effect on the spread or prevalence of CWD is unclear to date. Three studies evaluated this strategy using a deterministic predictive modeling approach [21, 23, 24]. In all models, the assumed mode of CWD transmission had a strong impact on the outcome. The mode of disease transmission is critically important in designing control strategies. In density-dependent (DD) transmission, the infection rate is determined by host density, where contact rates with infectious animals increase with increasing population size. In frequency-dependent (FD) transmission, the infection rate is independent of host density, i.e. overall population size, but is associated with contact in particular social groups [23, 36]. Disease spread can be reduced through population reduction if transmission is DD; however, population density control is less likely to be effective with FD transmission.

Wasserberg et al. [21] considered adaptive management as a framework for CWD control and developed a mathematical model to evaluate DD and FD transmission and how this impacts the effectiveness of various generalized deer culling. The authors used ‘harvest strategy’ to indicate recreational killing of deer for wildlife population management and ‘culling strategy’ for additional population reduction for disease management. However, both of these actions were generally achieved by changes in the recreational harvest rates of deer. If CWD transmission was FD, then disease prevalence could reach high levels and disease eradication might only be achieved by removing all deer in the infected population. If CWD transmission was DD, then disease prevalence could be controlled with lower impact on deer populations. Disease eradication might be achieved by reducing deer numbers with DD transmission. Moderate to high non-selective culling appeared to be an important tool for limiting the increase in CWD prevalence by removing infected deer and altering deer density regardless of the mode of transmission. The authors suggested that higher harvest rates could also be used to learn about managing CWD by producing prevalence patterns that distinguish DD transmission from FD transmission. High harvest rates should allow earlier detection of FD versus DD differences. Finally, the authors cautioned that culling strategies would need to be sustained over a sufficiently long period to evaluate their effectiveness.

The model presented by Potapov et al. [23] from the University of Alberta, using parameter estimates derived from data from white-tailed deer in the United States demonstrated that CWD eradication was possible through increased hunting alone under conditions of FD transmission assuming population recruitment or survival is DD. The compensatory population growth with healthy individuals would decrease CWD prevalence through dilution. Conversely, if CWD transmission was DD, harvest might increase disease transmission through a temporarily increased population size in situations where DD juvenile survival exceeded the removal of adults. The authors of that study point out that limited information regarding deer mortality rates and the impact of CWD on survival might have contributed to the uncertainty of their results and that their parameter estimates were only preliminary, especially with regards to prion persistence in the environment.

The third deterministic modeling study evaluated both non-selective removal and preferential removal of males and the findings are presented with the other papers summarizing selective removal [24]. For the non-selective harvest strategies, projected CWD prevalence increased to 26 %, while total post-harvest deer densities stabilized at <7 per km.

##### Analytical observational studies

Intensive non-selective culling was deemed effective in two of the three studies that empirically assessed this intervention [27, 28]. The first observational study conducted in Illinois by Mateus-Pinilla et al. [27] evaluated the effect of a multi-year non-selective deer removal strategy consisting of government culling, deer population control permits and nuisance deer removal permits (collectively termed ‘sharpshooting’) on CWD prevalence. The intervention pressure was categorized by frequency (duration of sharpshooting), effort (number of deer removed during intervention period) and intensity (average number of deer removed per year of sharpshooting). While a negative non-linear and non-monotonic association was identified between CWD prevalence and sharpshooting, the non-linear and non-monotonic effects of the intervention pressures on CWD prevalence varied by age and sex of deer. There was a more consistent association between population management programs and declining CWD prevalence in fawns and yearlings than in adult white-tailed deer. When measuring intervention pressure by frequency, the authors observed a significant decrease in CWD prevalence in fawns and yearlings when intervention was applied every year during the 5-year period from 2003 to 2007 (*P* < 0.001). Sharpshooting was most effective when 13–50 deer were removed during the 5-year intervention period (intensity); this effect was similar in fawns/yearlings (OR 0.33, 95 % confidence interval (CI) 0.14 to 0.76) compared to adults (OR 0.19, 95 % CI 0.06 to 0.60). Low-intensity sharpshooting (≤8 deer per year) and moderate intensity sharpshooting (28.1 – 59.0 deer per year) (compared to no sharpshooting) resulted in a significantly decreased CWD prevalence in males, and moderate intensity sharpshooting significantly reduced CWD prevalence in females.

Mateus-Pinilla et al. [27] identified a number of limitations that could have influenced their results. Sampling bias might have been introduced by selecting intervention management areas based on the existing CWD prevalence, i.e. selected areas were expected to have a greater overall CWD prevalence. Differences in passive and active surveillance may have affected the representativeness of the sampled population each year. The estimate of the frequency effect (duration of sharpshooting intervention in years) was imprecise because there was only a small number of management sections which implemented the intervention every year for the duration of the evaluation period. Furthermore, the evaluation period was relatively short and confined the study findings to a limited range of sharpshooting measures.

In the second observational study evaluating planned culling, Manjerovic et al. [28] compared CWD prevalence in Illinois and Wisconsin. When both states had an active government-organized culling program, CWD prevalence estimates did not differ significantly. However, when Wisconsin ceased government culling, CWD prevalence increased by 0.63 % annually, whereas CWD prevalence in Illinois remained unchanged. The authors concluded that government-organized planned culling could maintain low levels of CWD prevalence. The authors acknowledged that factors other than harvesting might have affected disease transmission and prevalence (e.g. forest cover; prion persistence) and contributed to differing prevalence between the two states. However, it was unlikely that such factors changed over time and could completely explain the temporal differences observed.

In the third study evaluating planned population control using ‘before-after-control-impact’ estimates of effect size in a case–control study design, Conner et al. [26] evaluated focal culling by sharpshooters in hot-spots in Colorado. Hot-spots were areas where surveillance data revealed a high CWD prevalence or case clusters which were then targeted by state wildlife management agency personnel for focal scale culling. This strategy did not result in a statistically significant difference in CWD prevalence between the treatment and control management areas (difference in prevalence 0.3, standard error 0.3). In addition to the possibility of true ineffectiveness of this intervention, the authors presented the potential for confounding as an alternative explanation for their results. Only data from male mule deer ≥1.3 years old were used due to known lower CWD prevalence in mule deer <1 year old. However, CWD prevalence in mule deer 5 to 7 years of age was 2.5× higher compared to 2 to 4 year old mule deer and 7.8× higher compared to yearlings. Therefore, authors concluded that different age-structures in the treatment- and control-management areas might have confounded the results of this study. Other reported study limitations were that the sample sizes in some of the evaluated management areas were small and that other concurrent management strategies might also have confounded results.

#### The effect of selective culling on CWD prevalence and deer population size

All studies evaluating selective culling used predictive models. The first study evaluated a ‘test-and-cull’ strategy through a stochastic model [20]. The effects of a ‘test-and-cull’ strategy were compared to deer populations without harvesting or CWD disease and two different CWD detection scenarios were investigated. In the first scenario, detection of CWD infection was assumed possible early in the course of the disease when deer were assumed to be only latently infected (latent infection was defined as having been exposed to an infective dose of the agent but not yet being able to transmit the disease). In the second scenario, infected deer were shedding the infective agent at a constant rate and were presumed to be clinically affected. Depending on the level and timing of testing the model predicted that this strategy effectively limited the rate of increase of CWD prevalence and eventually resulted in the elimination of the disease from most modeled populations. However, this was only effective in populations with low CWD prevalence (0.01 and 0.05), and where more than 80–90 % of infected deer were removed after 80 years of intervention. The effectiveness of the planned culling strategy was reduced dramatically when initial CWD population prevalence was modeled at 0.05 compared to 0.01, resulting in twice the time required to have a 50:50 chance of eliminating CWD from infected populations. Model projections suggested that CWD could become unmanageable if selective culling strategies were delayed.

The authors of this simulation study were unable to identify a set of realistic parameters that allowed sustained co-existence of deer populations and CWD [20]. In simulated deer populations left unmanaged, CWD-prevalence increased multiple-fold and resulted in deer extinction. Natural elimination of CWD in simulated populations occurred only if transmission was modeled at very low rates or when disease-decimated populations could recover because the few remaining diseased deer died before transmission occurred. Furthermore, the authors identified a number of sources of uncertainty with regards to model parameter estimates which they called “[…] at best a collection of educated guesses as biological mechanisms underlying CWD transmission are poorly understood […]”. They also noted that their model did not account for spatial attributes and patterns of geographic spread could not be assessed.

The role of predation in control of CWD was evaluated by Wild et al. [22] in a deterministic predictive model. Imposing non-selective mortality (any deer) from wolves decreased population size and CWD prevalence, resulting in persistence of CWD within the simulated population. Imposing selective mortality (where CWD-positive deer had 4× higher mortality) from wolves decreased population size more modestly while rapidly reducing CWD prevalence, resulting in disease elimination from a simulated closed population. Based on this model, CWD prevalence could be halved within a decade and eliminated within the century if a pack of wolves consistently and selectively removed 15 % of deer in a closed population. Furthermore, the model predicted that CWD emergence in new areas could be limited through selective predation. However, authors acknowledge that prey vulnerability, the nature of population compensation and factors affecting disease transmission greatly affect precise estimates of time required to attain results. The demonstrated beneficial effect of predation on CWD control was considered to be underestimated by the authors as they did not account for carcasses as a potential source of CWD. They concluded that carcasses infected with CWD would likely be removed by predators, thereby further decreasing the risk of CWD spread. The authors concluded that CWD prevalence consistently and robustly decreased in deer populations exposed to predation, and more so when exposed to selective predation.

In the final study using a deterministic compartmental model conducted by Jennelle et al. [24], sex-specific FD-transmission was best supported by the model which concluded that increased hunting pressure selecting for the sex-category with the greatest prevalence (males) reduced CWD prevalence while female- or herd-control-focused harvest increased CWD prevalence. Male-targeted harvest was predicted to result in a drop in CWD prevalence to < 5 % by 2060 and < 2.5 % by 2110. In that study, pure DD-transmission with equal sex infection coefficient was least supported by the data. The authors highlighted a number of limitations to their model design and results. Dispersion of CWD was assumed to occur uniformly from the point of origin and the possibility of movement over longer distances was ignored. Spatial heterogeneity and physical barriers to CWD dispersal were not considered in the model. The variance for the rate of CWD spread was likely higher than predicted because only six data points were considered in the rate of spread analysis. Furthermore, the authors specify that the model did not account for the potential of environmental transmission, differences in susceptibility due to Prnp genotype, or infectious contact with matrilineal social groups.

#### Seasonal (summer) hunting as an additional control strategy to regular hunter harvest

The last study to evaluate population control as a management strategy for CWD, and one which also considered disease transmission dynamics, was evaluated by Oraby et al. [25] using a deterministic predictive model. In this approach, the authors considered seasonal behaviour changes that could lead to a seasonal shift between DD and FD transmission in deer. As FD-transmission rate depends on social contact and behaviour, which differs between seasons, the disease transmission dynamics could vary seasonally. Therefore, the authors assumed a FD- and a DD-transmission mechanism in summer and winter, respectively, and evaluated different culling intensities (through different mortality rates) under these assumptions in both seasons. Overall, the model suggested that culling had beneficial effects on CWD transmission.

Based on their model assumptions, deer contact rate during the summer had a greater effect on R_0_ compared to contact rate during winter. The authors, therefore, suggested that culling should be concentrated in the summer provided costs between summer and winter culling were comparable. However, similar to the findings by Gross and Miller [20], careful consideration would have to be given to culling rates to ensure survival of the herd as summer culling was limited by the need to preserve the herd and authors cautioned that summer culling might be more difficult given that deer herds roam in a larger area compared to winter. It is also important to recognize that the results by Oraby et al. [25] were affected by their choice of modeling disease transmission as FD in the summer. By doing so, the effect that the geographical spread of the herd had on R_0_ in winter and summer cancelled out in the model. Additionally, the model also showed that increasing the duration of the summer season would increase R_0_ and make CWD eradication more difficult.

#### Vaccination

Potapov et al. [23] also considered the potential effectiveness of vaccination in managing CWD in their deterministic model. Although the model suggested vaccination could be effective if a vaccination strategy was applied simultaneously to harvesting, the proportion of the population that is vaccinated might have to increase to achieve disease eradication as harvest could enhance disease spread by removing immune individuals.

### Sources of uncertainty and limitations

All studies identified at least some areas of uncertainty or bias and other limitations that might have affected study results. An additional summary table of evidence for effectiveness, sources of uncertainty, and other limitations in these studies is shown in more detail (see Additional file 3). A major source of uncertainty identified in four studies [21, 23–25] and alluded to in a fifth [22] was the relative contributions of different modes, FD or DD, of CWD transmission. Other sources of uncertainty affecting CWD transmission, such as prion dynamics in the environment, were specifically identified in two studies [23, 28] and alluded to in a third [22].

Similarly, Jennelle et al. [24] specifically commented that their model did not account for indirect transmission from the environment in which prions can survive for years. Only two of the six papers using predictive modeling and addressing the effectiveness of control measures included the potential for indirect or environmental transmission in the models [22, 23].

Sampling bias was identified as a potential limitation in a number of studies evaluated here. Sources of sampling bias included selective surveillance sampling, for example focused on specific age or sex groups; sampling based on geographic sections with high CWD prevalence; different sampling populations derived from passive versus active surveillance; and sampling during only one season of the year [24, 26, 27].

The most common source of uncertainty identified by authors conducting predictive modeling studies was model parameter estimates. Wild et al. [22] mentioned that uncertainty surrounding parameter estimates limited the confidence in predicting the exact time frames required for disease control or elimination in their study. Gross and Miller [20] called their parameter estimates ‘at best a collection of educated guesses’ and in their case, the lack of data over time also limited their ability to verify model projections. Potapov et al. [23] pointed out that their parameter estimates were only preliminary and that using different parameter estimates resulted in different conclusions. However, all of the studies reported at least some analysis of the sensitivity of the model to different parameter estimates. One of the models included differences in contact rates or transmission probabilities based on sex [24], but none of the models considered differences in contact or transmission probabilities based on age. Three of the six modeling studies [20, 21, 25] incorporated differences in model parameters based on season, with one study specifically examining differences in behavior and impact on disease transmission [25].

### Knowledge gaps identified by study authors

Many of the uncertainties and study limitations identified by the authors of the articles reviewed here stem from knowledge gaps with regards to CWD epidemiology and, therefore, challenge the development of effective control strategies. Various aspects surrounding the uncertainty about disease transmission dynamics, such as disease transmission coefficients, the drivers for spatial dispersion, seasonal influences on transmission rates and survival of prion in the environment and, therefore, risk of indirect transmission, were highlighted in a number of studies [20, 24, 25]. Jennelle et al. [24] also emphasized the knowledge gap in the differences in infectious contact between and among sexes. The lack of practical and reliable tests to detect pre- or subclinical CWD in the field was further identified as an important area of future research [20]. Absence of such tests impedes early detection of CWD in a population and, therefore, also evaluation of the effectiveness of control programs.

## Discussion

This systematic review of field-applicable strategies for the control of CWD in wild deer in North America identified nine individual articles evaluating four unique interventions. Six of the nine studies were predictive models; only three were based on observational data. The evidence identified in the current peer-reviewed literature was not sufficient to clearly support the recommendation of a primary control strategy for CWD. This review not only highlights the limited amount of information available regarding field-tested CWD interventions, but also the knowledge gaps that exists with regard to control efforts for CWD.

No studies were identified that directly examined the efficacy of control options other than population management. There was evidence that increasing general overall hunting pressure might be effective for CWD control but additional intensive culling by sharp-shooting and other means was the only intervention that empirically appeared to control CWD. Based on one of the predictive modeling studies, selective culling of males through hunting should be more effective than a general increase in hunting pressure alone and might be a means of improving CWD control through hunting. Additionally, selective or preferential removal of CWD infected deer was shown to be effective in reducing CWD prevalence. One study evaluated this in the context of predation while another evaluated a test and cull strategy. Recent evidence that CWD infected deer are more likely to be shot by hunters suggests hunting may also selectively remove infected deer and be more effective in controlling CWD than predictive models would indicate [37].

The strength of the evidence presented in the nine articles must be put in context of the respective study designs and associated limitations. No study evaluated an intervention using a randomized controlled trial (RCT), generally considered to give the best quality of evidence for evaluating an intervention. There are several factors that impact the feasibility of RCT for CWD field research including: the long and variable incubation period (18 months – 5 years); the absence of a rapid, practical and reliable ante-mortem field tests for detecting preclinical CWD; the limited understanding of transmission dynamics under different field conditions; factors affecting prion survival in the environment and the risk of indirect transmission; the challenge of a suitable control group; vast geographic dispersal of deer populations; and different social behaviours between seasons and between age- and sex-groups which might affect transmission rates.

Given the limited feasibility of large scale randomized-controlled studies, the two best alternatives were dynamic simulation models and observational studies. The limitations of the existing studies need to be considered separately recognizing the inherent differences between dynamic models and observational studies. Most studies were based on deterministic predictive modeling where outcomes were fully determined by the assumed conditions and selected parameters. The deterministic modeling studies, therefore, did not account for inherent randomness in infection transmission dynamics [21–25]. Results derived from predictive modeling studies depend on the accuracy and precision of the estimated parameters and on the scope of parameters included in the model. Therefore, the results were only predictive within the set of assumed parameters. However, all of the modeling studies included some analysis exploring the sensitivity of the results to alternative parameter values. For example, the potential for predation to decrease CWD prevalence was reported by Wild et al. [22] using a deterministic model. However, the findings of the study were supported with a sensitivity analysis that indicated a consistent trend in decreasing CWD prevalence over a range of parameter estimates and within different deer population structures.

Given the known complexity of CWD epidemiology, the stochasticity of disease transmission, and identified knowledge gaps, the usefulness of extrapolating results of predictive models must be carefully considered. Only the model approach by Gross and Miller [20] was agent-based and stochastic. The potential for differential mixing and disease transmission based on sex was accounted for in 1 paper [24], and none of the models accounted for differential mixing and transmission based on age. Similarly, the potential for environmental transmission was only accounted for in two studies [22, 23] and seasonal effects were only considered in three of the six studies [20, 21, 25]. This discussion highlights the variability in model structure and parameter assumptions and echoes the challenges regarding the most appropriate CWD transmission coefficient also encountered by other authors including Jennelle et al. [24] and Potapov et al. [23].

Attempt to better capture the complexity of CWD transmission in dynamic modeling studies continue. At the time this manuscript was being submitted for consideration, a new modeling paper was published which examined the potential for different harvest management policies to reduce CWD prevalence while minimizing impact on population stability [38]. The model considered four age and gender groups that could be harvested at different rates and which experienced different forces of infection under FD transmission dynamics resulting from both environmental and direct contact mechanisms. Harvest management policies that focussed on male deer reduced the prevalence of CWD most consistently across different disease transmission mechanisms.

The remaining studies assessed in this review were analytical, observational studies, all of which evaluated planned culling for the control of CWD. One of the primary outcomes determined in all three studies was CWD prevalence derived from post-harvest testing of hunted and government culled deer. Selection bias and misinterpretation of the true prevalence of CWD could result in these studies from differences in CWD prevalence between harvested and non-harvested samples, as well as from differences in active and passive surveillance [27]. Mateus-Pinilla et al. [27] considered that selection bias could also be introduced by selecting study areas with a known high CWD prevalence. Similarly, Conner et al. [26] highlighted that spatial heterogeneity might affect CWD prevalence or that confounding could have occurred from alternative management strategies applied to some of their study areas. Other potential confounders identified in all three studies were differences in environmental measures and topography that might have affected deer abundance and CWD prevalence or differences in active and passive surveillance across the study areas and over time [26–28].

Any control program must consider its stakeholders and management strategies associated with deer culling must take into account public acceptance and hunters’ attitudes and behaviours. Public opinion, societal acceptance and economic considerations will influence the ultimate strategy. Culling of wildlife is often unpopular but can be a necessary method to preserve a population or limit the spread of a disease. This poses a challenge for responsible regulatory agencies as they may have to carefully navigate public acceptance of the control strategy to ensure continued public support for wildlife management.

Hunter harvest does not explicitly target high CWD prevalence areas [28]. Cooney and Holsman [39] found a relatively strong relationship between hunters’ CWD risk perceptions and efficacy of a management strategy involving deer density reductions. Hunters surveyed did not believe that hunters could effectively reduce deer populations or eradicate CWD. Similarly, based on survey data from hunters and landowners in Wisconsin, Holsman et al. [40] highlighted specific psychological reasons for hunter opposition to the deer reduction strategies implemented in that state and concluded that due to hunter opposition, recreational hunting was unlikely a successful approach to significant deer population reduction.

These psychological factors must be considered when developing any intervention program yet they have not been included in predictive modeling studies evaluating increased hunting pressure [21, 23, 24]. The other major challenge identified by two of the three studies was uncertainty about the disease transmission coefficient, which affected interpretation of the results and made definitive conclusion about increasing hunting pressure as an effective intervention difficult. In combination, these may in part also be the reasons why planned culling approaches to date appear to have more evidence supporting their effectiveness in CWD control in wild deer populations. Due to the more selective nature of the intervention public acceptance might more easily achieved.

Interestingly, Holsman et al. [40] concluded that trap, test and cull strategies would likely find more public/hunter support than unselective culling. One study in this review here evaluated this selective culling approach [20]. Besides the limitations regarding parameter estimates for the model applied by the authors of that study, a major drawback of test-and-cull strategies is the current lack of practical live-animal tests for early disease detection. Tonsil and rectal biopsies have shown some utility but current research is focusing on blood, saliva, urine, fecal and nasal brush samples [15, 41, 42]. However, even if more applicable ante-mortem tests become available, logistics and costs will remain challenging to testing and culling in the field. Consideration must also be given to the potential dispersal distances of infected deer which is not limited by political boundaries. Regional and interstate/interprovincial cooperation and collaboration will be an important part of any successful management strategy.

The evidence in support of predator species for the control of CWD is worth consideration, and is not only limited to the study by Wild et al. [22] evaluated here. Deer killed by mountain lions in Colorado had significantly higher CWD prevalence compared to hunter-killed deer, suggesting that mountain lions selectively preyed on infected deer [43]. Similarly, mountain lion attacks against CWD-infected deer were increased fourfold compared to non-infected deer [44]. Decreased attentiveness of CWD-infected mule deer was considered the reason for their greater propensity to vehicle collisions [43]. If predators are able to detect a subtle difference in CWD-infected deer early in the course of the disease, removal of these deer by predators may be more effective at decreasing the spread of CWD compared to non-selective culling. Furthermore, it was suggested that the absence of large predators could pose a risk factor for the establishment of CWD in new areas [22].

Wild et al. [22] highlighted additional hypothesized benefits of predator species to the CWD control. Consumption of potentially infective carcasses might contribute to the removal of infective tissue from the environment and passage of prion proteins through the alimentary tract of predator species could potentially reduce their infectivity. Additionally, deer behaviour could change in response to the presence of predators resulting in changes in land use and habitat preferences, thereby potentially diminishing contact rates with infective environmental contaminants. The maintenance and in some cases introduction of large predators into CWD-infected areas would need public approval. While the introduction or increased protection for predator populations is highly contentious especially in ranching country, natural predation of CWD-infected deer might be more acceptable to some public interest groups compared to non-selective deer culling.

Other factors that could inform decision making with regards to CWD control must be further investigated and considered in future control strategies. These include but are not limited to gaining a better understanding of the importance of indirect (environmental) transmission; prion survival in the environment (soil binding; plant uptake; water contamination); practical ante-mortem testing; and preventive (vaccine) strategies. Although studies are available that investigated aspects of these important questions concerning CWD, knowledge about these factors is currently insufficient to fully understand how they might be directly applied in CWD control.

Research is needed to optimize policies for CWD control in wild deer, particularly for mule deer populations that predominate in many affected areas, particularly in Canada. Most of the current CWD management studies were conducted on white-tailed deer. Mule deer differ from white-tailed deer with regards to their social behaviours and habitats yet there is currently insufficient evidence to know whether different management strategies are necessary for white-tailed and mule deer. Despite the apparent gaps in the available information, the weight of evidence from a recent long-term study of CWD in mule deer populations in Colorado suggested that the CWD outbreak might be declining over time and that the decline might be related to population control efforts [45]. The population of CWD infected deer was considered to be stable. This study suggests that long-term management of CWD in wild deer is possible and that efforts to control CWD in Canada are justified.

This systematic review has a number of limitations that warrant acknowledgment. The search was restricted to articles from North America; however, this was unlikely to be an important limitation as CWD to date has only been reported from this continent, the Republic of Korea, and in Sweden in early 2016. It follows that most relevant research has been from North America although it is conceivable that relevant literature from other parts of the world might have been missed. Similarly, only articles published in English were included and no translations were conducted of the literature published in other languages. The review was restricted to the peer-reviewed literature although a quick survey of the grey-literature did not identify any additional new information.

The search focused on evaluations of field-applicable control strategies for CWD; therefore, experimental studies or in vitro assays investigating prevention and control that were in the early stages of development, e.g. vaccination or therapies, were excluded. Although such research has potential relevance in future management policies for CWD, information on the implementation, feasibility and efficacy of applying these management strategies in field situations is currently lacking. Therefore, these exploratory studies were only briefly reported here. Similarly, many studies investigated risk factors for CWD that could potentially be manipulated in some way to influence control. Studies that did not explicitly evaluate the impact of an intervention on CWD in wild deer populations were not included despite potentially containing information that might inform CWD management. Lastly, this review did not include approaches to control of CWD in farmed deer. Because of the complexity of managing this disease in wildlife populations and the existing knowledge gaps about CWD transmission and prion survivability in the environment, the focus on disease control in wild populations under field conditions was of specific interest.

Given the substantial number of limitations in the existing data and additional research needs identified in this study, there is a strong case for the utility of expert elicitation and tools such as multi-criteria decision analysis to identify the uncertainties that are most important to resolve in order to best achieve management objectives for CWD [46]. While additional information is being collected, adaptive management, a structured decision making approach to solving dynamic problems, has been proposed as a tool for optimizing intervention options in the face of scientific uncertainty [47]. The findings of this study support the need for ongoing real-time surveillance in conjunction with cervid demographic studies that can be used to inform adaptive management, decrease uncertainty and ultimately improve the control of CWD.

## Conclusions

This systematic review highlighted the paucity of proven control strategies for CWD in wild deer populations in North America. Although studies using predictive models demonstrate the potential for increased hunting pressure, intensive culling, selective removal of infected deer, and summer hunting to be effective in managing CWD, these studies are limited by the incomplete understanding of transmission dynamics. The feasibility of implementation of these strategies over the timelines of decades as proposed, is also unclear. Only intensive non-selective culling using sharp-shooters has support from observational studies. Additional analytical field studies and studies of management strategies other than population control are needed. Important knowledge gaps are evident and CWD research is challenged by the long incubation period, the lack of reliable ante-mortem testing, the complexity of a disease surveillance and management in wildlife populations, and the hardiness of the infective prion protein in the environment. Wildlife management agencies face the difficult task of using the currently limited evidence to inform decision making for CWD control programs. The challenge to evaluate the effectiveness of current and emerging control strategies under field conditions remains for CWD researchers.

## Additional files

Additional file 1: PRISMA 2009 checklist. The items of the checklist pertain to the content of this systematic review which includes title, abstract, methods, results, discussion and funding. (DOC 67 kb) Additional file 2: Table with number of unique articles collected from each database searched for literature on management strategies to control chronic wasting disease in wild deer in North America. (DOCX 26 kb) Additional file 3: Summary table of evidence for effectiveness, sources of uncertainty, and other limitations for 9 predictive and observational studies evaluating control of chronic wasting disease in wild deer in North America. (DOCX 36 kb)
