# Supplementary material for: Systematic review of management strategies to control chronic wasting disease in wild deer populations in North America
Source: BMC Vet Res. 2016 Aug 22;12(1):173. doi: 10.1186/s12917-016-0804-7 (PMC4994292; doi:10.1186/s12917-016-0804-7)
Supplement: Additional file 2: — Table with number of unique articles collected from each database searched for literature on management strategies to control chronic wasting disease in wild deer in North America. (DOCX 26 kb) [file 12917_2016_804_MOESM2_ESM.docx]

Additional file 2. Number of unique articles collected from each database searched for literature on management strategies to control chronic wasting disease in wild deer in North America.

| Platform | Database name | # articles exported to EndNote^®^ | # articles after deduplication within the database | # unique articles after deduplication between all databases |
| --- | --- | --- | --- | --- |
| Ovid | Embase Classic & Embase [1947- 2015 week 19] | 304 | 300 | 151 |
|  | MEDLINE | 272 | 267 | 175 |
| Web of Science | CABI: CAB Abstracts and Global Health | 248 | 248 | 108 |
|  | BIOSIS Citation Index | 202 | 202 | 57 |
|  | Zoological Record | 155 | 155 | 53 |
|  | Web of Science Core Collection | 307 | 306 | 152 |
| ProQuest | AGRICOLA | 123 | 123 | 39 |
|  | BioOne Abstracts & Indexes | 17 | 17 | 16 |
|  | Animal Behavior Abstracts | 10 | 10 | 6 |
| Scopus | Scopus | 217 | 217 | 42 |
| Total |  | 1855 | 1845 | 799 |
