# Supplementary material for: Systematic review of management strategies to control chronic wasting disease in wild deer populations in North America
Source: BMC Vet Res. 2016 Aug 22;12(1):173. doi: 10.1186/s12917-016-0804-7 (PMC4994292; doi:10.1186/s12917-016-0804-7)
Supplement: Additional file 3: — Summary table of evidence for effectiveness, sources of uncertainty, and other limitations for 9 predictive and observational studies evaluating control of chronic wasting disease in wild deer in North America. (DOCX 36 kb) [file 12917_2016_804_MOESM3_ESM.docx]

Additional file 3. Summary of evidence for effectiveness, sources of uncertainty, and other limitations for 9 predictive and observational studies evaluating control of chronic wasting disease in wild deer in North America.

| **Citation** | **Outcome result/evidence for effectiveness** | **Critical assumptions and sources of uncertainty in predictive modeling studies or source of bias and error in descriptive/analytical studies** |
| --- | --- | --- |
| **Predictive modeling studies** |  |  |
| Gross JE, Miller MW. Chronic wasting disease in mule deer: disease dynamics and control. Journal of Wildlife Management 2001;65:205-215 [20] | Authors were unable to identify set of realistic parameters that allowed sustained co-existence of deer populations and CWD  In simulated deer populations left unmanaged, CWD-prevalence would increase multiple-fold and result in deer extinction  Simulated selective culling (<20% of infective population) was effective only if initiated when disease prevalence was very low (<0.01). Likelihood of success diminished rapidly with increased prevalence. | 1. Model mechanisms described by the authors as “at best a collection of educated guesses” as biological mechanisms underlying CWD transmission were poorly understood.  2. Lack of independent, replicate parameter estimates and data on prevalence trends over time also limited researchers' ability to verify model projections. Researchers conclude that they have much greater faith in short-term projections than long-term projections.  3. Model did not include spatial attributes; therefore, the potential for and patterns of geographic spread cannot be assessed.  Authors reiterate that the lack of including spatial attributes was mostly by design: the model described in this study is the core for a fully spatial model that the authors were developing at the time and which was to be used to evaluate large-scale control measures and design adaptive management strategies for limiting distribution and prevalence of CWD in free-ranging populations  4. The model did not account for environmental transmission. |
| Wasserberg G, Osnas EE, Rolley RE, Samuel MD. Host culling as an adaptive management tool for chronic wasting disease in white-tailed deer: a modelling study. Journal of Applied Ecology 2009;46:457-466 [21] | If CWD transmission is FD, then disease prevalence could reach high levels and disease eradication might only be achieved by complete population culling.  If CWD transmission is DD, then disease prevalence could be controlled with minimal impact on deer populations.  Disease eradication might be achieved by reducing deer numbers.  Deer culling appears to be an important tool for limiting the increase in CWD prevalence by removing infected deer and altering deer density.  Higher harvest rates can also be used to learn about managing CWD by producing prevalence patterns that distinguish DD transmission from FD transmission.  High harvest rates allow earlier detection of FD vs DD differences.  Culling strategies must be sustained over a sufficiently long period to evaluate their effectiveness. | 1. There is considerable controversy about the appropriate management actions to control CWD, primarily because FD vs. DD transmission and the long-term impacts on white-tailed deer populations are unknown.  2. There was uncertainty in the presentation of the results regarding the differences between the potential effectiveness of increased hunting pressure as compared to planned culling.  3. The model did not account for environmental transmission |
| Wild MA, Hobbs NT, Graham MS, Miller MW. The role of predation in disease control: a comparison of selective and nonselective removal on prion disease dynamics in deer. J Wildl Dis 2011;47:78-93 [22] | Predictive model suggests the disease control measure is effective  Imposing non-selective mortality (any deer) from wolves decreased population size and CWD prevalence, resulting in persistence of CWD within the simulated population.  Imposing selective mortality (CWD-positive deer 4X increased risk) from wolves decreased population size more modestly while rapidly reducing CWD prevalence, resulting in disease elimination from a simulated closed population.  Trend was robust over a range of parameter estimates. | 1. Uncertainty in parameter estimates limited confidence in predicting exact timeframe required for disease control or elimination.  For example, the authors assumed that transmission rates were approximately 25 times lower in natural populations than in captive ones.  2. Authors acknowledged that prey vulnerability, the nature of population compensation and factors affecting disease transmission greatly affect precise estimates of time required to attain results.  3. Predation intensity was assumed to not change with prey abundance.  4. Model did not account for the age structure of the population. |
| Potapov A, Merrill E, Lewis MA. Wildlife disease elimination and density dependence. Proc Biol Sci 2012;279:3139-3145 [23] | Using a mathematical model, the authors reported that culling or harvesting could eradicate CWD, even when transmission dynamics were FD. Eradication can be achieved under FD transmission with DD birth or recruitment of new, healthy individuals, which reduced disease prevalence by dilution.  Effectiveness of evaluated control measures depends on the disease transmission coefficient; while immunization may be effective to stop spread of CWD assuming FD^$^ transmission, if harvesting occurs at the same time, vaccination efforts may have to increase to achieve disease eradication due to loss of vaccinated deer. | 1. Limited information on deer mortality might have contributed to uncertainty in results; the survival rate for deer and increase in mortality associate with CWD infection were extrapolated from different regions.  2. The parameter estimates used were only preliminary, particularly with regards to prion dynamics in the environment and their accessibility over time. Using different parameter estimates resulted in different conclusions.  3. Only 3 adult age classes considered: susceptible, infected, and immune. |
| Jennelle CS, Henaux V, Wasserberg G, Thiagarajan B, Rolley RE, Samuel MD. Transmission of Chronic Wasting Disease in Wisconsin White-Tailed Deer: Implications for Disease Spread and Management. PLoS ONE 2014;9:e9104 [24] | Harvest focused on male deer could result in stable population dynamics and control of CWD within 50 years, given the constraints of the model.  Male-focused harvest resulted in declining CWD prevalence resulting in a female-dominated population structure.  Herd-control or female-focused harvest projected increased CWD-prevalence. | 1. Estimated spread assumed as a uniform diffusion from point of origin and ignored potential disease movement via longer distance dispersal.  2. Analysis does not account for habitat heterogeneity and physical barriers.  3. Despite high R^2^ values, regression only uses 6 data points to evaluate spread, therefore, there's likely a higher variance associated with the estimated rate of spread. Although authors attempted to account for over dispersion, estimates might be too precise as they did not account explicitly for lack of independence and/or autocorrelation.  4. The model does not account for environmental transmission, PrnP genotype, or infectious contact with matrilineal groups.  5. Data were collected every year within one season [winter] only; therefore, estimates of seasonal, environmental, or between/among sex based on assumptions about the infectious contact structure. |
| Oraby T, Vasilyeva O, Krewski D, Lutscher F. Modeling seasonal behavior changes and disease transmission with application to chronic wasting disease. J Theor Biol 2014;340:50-59 [25] | Predictive model suggests the disease control measure was effective.  The authors allow for differences in DD or FD in different seasons.  Deer contact rate during the summer season has greater effect on R_0_ than contact rate during winter season. Summer culling was an effective disease eradication strategy but was limited by the need to preserve the herd.  The model was parameterized from data derived from the scientific literature on CWD and deer ecology. | 1. This model does not account for indirect or environmental transmission. The authors discuss the complexity of adding environmental transmission to the model.  2. The model does not account for the age or sex structure of the population.  3. The authors discussed extending the model to consider more than 2 seasons and specifically include rut. |
| **Analytical observational studies** |  |  |
| Conner MM, Miller MW, Ebinger MR, Burnham KP. A meta-BACI approach for evaluating management intervention on chronic wasting disease in mule deer. Ecological applications : a publication of the Ecological Society of America 2007;17:140-153 [26] | Areas where surveillance data suggested a high CWD prevalence or case clusters were targeted by state wildlife management agency personnel for focal scale (on average <17 km^2^) culling, in most cases using agency sharpshooters.  Treatment areas, along with spatially paired control areas were constructed post hoc in a case-control design and delineated using home range estimators.  Control measure did not result in a statistically significant change in CWD prevalence | 1. The authors stated that treatment might not have been adequate or the timeframe too short for a treatment effect to be detected. Deer removal was inconsistent among sites, but regression analysis did not find an association between numbers of deer removed or prevalence and effect size.  2. The authors recognized the potential for confounding by differential distribution of older male deer between case and control regions.  3. There was also potential confounding by concurrent alternative management strategies that changed over the course of the study.  4. The authors also reported limited sample sizes in some management evaluation sites or limited time to see an effect of management changes. |
| Mateus-Pinilla N, Weng H-Y, Ruiz MO, Shelton P, Novakofski J. Evaluation of a wild white-tailed deer population management program for controlling chronic wasting disease in Illinois, 2003-2008. Prev Vet Med 2013;110:541-548 [27] | Control measure resulted in a statistically significant change in CWD prevalence.^#^  The strength of the association varied depending on age of the deer and the measure of intervention pressure.  The intervention consisted of measures of deer removal from three deer population control programs: Illinois Department of Natural Resources culling, deer population control permits and nuisance deer removal permits. The analysis included 14,650 white-tailed deer CWD test results with location and demographic data collected from both deer harvested in the interventions, as well as deer from hunter harvests and vehicle collisions. | 1. There was a potential for selection bias. The selection of geographic sections for intervention was dependent on CWD prevalence which were expected to be higher than overall prevalence.  2. Passive and active surveillance may not provide equally representative samples of the population each year.  3. The estimated association between sharpshooting and change in CWD prevalence could have biased due to residual confounding (even though they controlled for confounding effects of the environmental measures that were known to be related to deer population densities).  4. The study period was relatively short which confined study findings to a limited range of sharpshooting measures. |
| Manjerovic MB, Green ML, Mateus-Pinilla N, Novakofski J. The importance of localized culling in stabilizing chronic wasting disease prevalence in white-tailed deer populations. Prev Vet Med 2014;113:139-145 [28] | Removal of control measure resulted in a continuous increase in CWD prevalence and continuation of control measure resulted in stable CWD prevalence over time.  Researchers evaluated 10 years of CWD test results from Illinois and Wisconsin. | 1. The authors recognize that factors other than hunting might have affected disease transmission and prevalence [e.g. forest cover; prion persistence/soil composition] and this could contribute to differing prevalence between the two States in which control intervention was evaluated, these factors were not considered sufficient to explain the temporal differences observed. |

^#^while control measure was effective in all deer populations analyzed in this study, the magnitude of the effect varied by age (fawns, yearlings vs. adults) and by sex and depended on harvest intensity, frequency and effort.

^$^FD (frequency-dependent)
